# A comparison of the performance on extrinsic and intrinsic cartographic visualizations through correctness, response time and cognitive processing

**DOI:** 10.1371/journal.pone.0250164

**Published:** 2021-04-21

**Authors:** Čeněk Šašinka, Zdeněk Stachoň, Jiří Čeněk, Alžběta Šašinková, Stanislav Popelka, Pavel Ugwitz, David Lacko

**Affiliations:** 1 Department of Information and Library Studies, Faculty of Arts, Masaryk University, Brno, Czech Republic; 2 Department of Geography, Faculty of Science, Masaryk University, Brno, Czech Republic; 3 Department of Geoinformatics, Faculty of Science, Palacký University Olomouc, Olomouc, Czech Republic; University of Wisconsin Madison, UNITED STATES

## Abstract

The aim of this study was to compare the performance of two bivariate visualizations by measuring response correctness (error rate) and response time, and to identify the differences in cognitive processes involved in map-reading tasks by using eye-tracking methods. The present study is based on our previous research and the hypothesis that the use of different visualization methods may lead to significant cognitive-processing differences. We applied extrinsic and intrinsic visualizations in the study. Participants in the experiment were presented maps which depicted two variables (soil moisture and soil depth) and asked to identify the areas which displayed either a single condition (e.g., “find an area with low soil depth”) or both conditions (e.g., “find an area with high soil moisture *and* low soil depth”). The research sample was composed of 31 social sciences and humanities university students. The experiment was performed under laboratory conditions, and Hypothesis software was used for data collection. Eye-tracking data were collected for 23 of the participants. An SMI RED-m eye-tracker was used to determine whether either of the two visualization methods was more efficient for solving the given map-reading tasks. Our results showed that with the intrinsic visualization method, the participants spent significantly more time with the map legend. This result suggests that extrinsic and intrinsic visualizations induce different cognitive processes. The intrinsic method was observed to generally require more time and led to higher error rates. In summary, the extrinsic method was found to be more efficient than the intrinsic method, although the difference was less pronounced in the tasks which contained two variables, which proved to be better suited to intrinsic visualization.

## Introduction

The awareness that maps serve as tools for the creation of mental representations of the world and cannot therefore be considered transparent or direct depictions has long been discussed in cartography [1]. As a research topic, the cognition of maps is rooted in the early twentieth century [2]. A key question is how a particular form of cartographic visualization affects the effectiveness of cartographic communication [3–5]. The same data can be represented by different cartographic visualization methods. An unsuitable method not only reduces performance but also places various requirements which correspond to the type of cartographic visualization on different types of users and tasks [6, 7]. User characteristics (such as cartographic skills, [8, 9]) and the type of task [10, 11] must therefore be considered when we conduct empirical studies on the performance of alternative visualizations.

**Fig 1:**
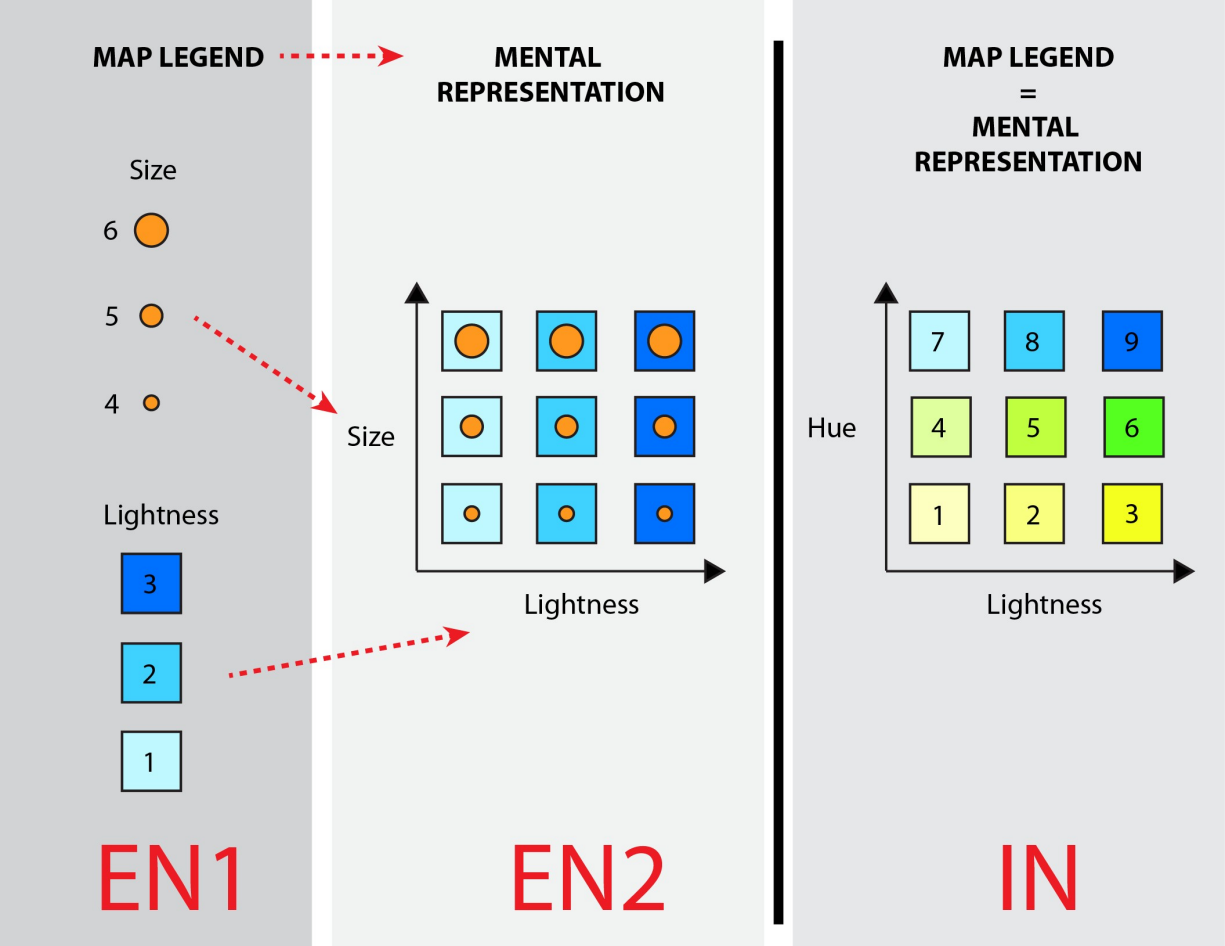
Examples of extrinsic and intrinsic bivariate encoding of geographic variables. EN1: extrinsic (separable) encoding of variables according to size and color lightness; EN2: mental representation of extrinsic visualization (all the possibilities); IN: intrinsic (inseparable) encoding of variables according to hue and lightness.

The primary aim of the present study was an empirical and objective comparison of two alternative bivariate visualizations (Fig 1) to assess the performance of a selected population which possessed a basic level of cartographic skill [12–16] through two different types of task. Another aim was to understand the cognitive processes which underlie the potential differences in objective performance [17–20].

Olson [21] stressed that maps are considered highly valuable visual stimuli in experimental psychology since the variables they represent can be accurately controlled. The manner of presenting geographic information can have a significant effect on user cognitive processing (internal mental processes) during map-related tasks. Larkin and Simon [22] presented the concept of informational and computational equivalence and argued that different visualizations can be informationally equivalent if all the information available in one of them is available in the other, and vice versa. The establishment of informational equivalence between bivariate cartographic visualizations permits us to investigate the extent of computational equivalence between the two.

Cartographic visualization offers numerous methods of presenting geographical data. These methods differ in their ability to visualize certain data types, the level of detail they provide, and the number of variables they simultaneously portray [23]. The graphic display of multiple geographic phenomena is known as multivariate mapping [24], and its purpose is to investigate the relationships between the given phenomena. Bivariate maps encode two separate variables simultaneously [25]. Bivariate mapping can be further divided into extrinsic (the variables carrying the information are visually separable) and intrinsic (the variables are visually inseparable [26]).

The present study applies both extrinsic and intrinsic bivariate encoding of geographic variables (Fig 1) to investigate the cognitive processes of map users.

The extrinsic bivariate method employs two visually distinct variables (the differences may represent, for example, size, shape or color lightness) to display two different geographical phenomena, such as soil depth and moisture. In the present study, each of the two phenomena had three levels of intensity (low, medium and high), which provided a total of six options in the map legend (Fig 1, EN1 left). From the map legend, the map users were required to create a mental representation of nine possible combinations (Fig 1, EN2 center). Intrinsic bivariate visualizations apply visual variables which are visually inseparable (typically, the visual variables include hue, color lightness and opacity), resulting in a map legend comprising nine combinations (Fig 1, IN right). In this latter case, the map legend was identical to the mental representation of all the possible combinations. Although, color lightness, hue and opacity are considered to interact with each other in the psychology of perception [27–29], cartography regards them as mutually independent entities [30]. Therefore, in cartography, these parameters are used as independent visual variables.

Each visualization type can be expected to induce a different cognitive and perceptual load on the user [31–33]. The differences in cognitive processing relate to selective attention theory, which specifies that only a limited number of elements can be processed at one time [34]. The perception aspect can be explained according to pre-attentive visual processing theory [35, 36]. Some visual elements, designated pre-attentive, can be detected in a single glance and thereby serve as the central components of a visualization. In map reading, pre-attentive elements can aid in identifying boundaries and detecting the presence or absence of other elements; for example, size (an extrinsic variable) is pre-attentive, while lightness (an intrinsic variable) cannot be considered a pre-attentive feature. Since the processing of extrinsic and intrinsic visual elements is not only based on perception but involves a broader cognitive context, it appears reasonable to assume that the situation will be more complex when both extrinsic and intrinsic visual variables are employed.

Bivariate mapping and the use of various visual variable combinations have been the subject of numerous research studies [e.g., 21, 37–42]. Elmer conducted an extensive comparison of visual variable combinations [43]. Kunz studied the use of bivariate visualization methods (extrinsic and intrinsic) to produce visualizations of natural hazards (avalanches) and the levels of uncertainty in the presented data (avalanche hazard prediction) [44]. Šašinka et al. investigated the differences in processing intrinsic and extrinsic visualizations, focusing mainly on cognitive style and map reading skills [45]. The results of the study (and of related eye-tracking studies) revealed significant differences between extrinsic and intrinsic visualizations associated with both map-reading performance and task processing. Although the aim of the study was not to compare methods of visualization, the results showed that a group of laypersons (psychology students) worked more effectively with the extrinsic method, while participants with better map reading skills (cartography students) demonstrated better performance using intrinsic visualization.

However, the authors noted an important limitation in their study, consisting in a relatively sophisticated topic (avalanche risk and its uncertainty) which the participants (especially psychology students) may have found difficult to understand.

**Fig 2:**
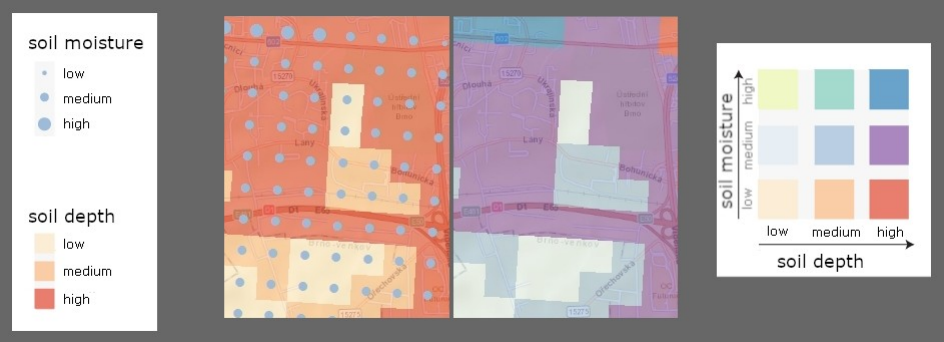
**Examples of extrinsic visualization (left) and intrinsic visualization (right) used in the study.** Legends for both extrinsic and intrinsic visualization are also given. Areas with identical values are depicted with two different encoding systems to enable a visual comparison of the differences between each visualization.

The present study was designed based on the results of the above studies. The stimulus material included two bivariate maps with the same content; an example is shown in Fig 2. We selected soil depth and soil moisture as suitable phenomena for depiction since they are considered generally comprehensible and quantifiable. Information gathered from volunteers informed our selection of the topic during the experiment design process. It was critical that participants intuitively understood the relationship between the visual variables and the depicted phenomena in the legend’s design. We followed the principle of cultural metaphors and applied a visual representation of the data to match the metaphors which aid conceptual thinking [6, 46]. We used a combination of color and size for extrinsic visualizations and different colors for intrinsic visualizations. Fig 2 illustrates examples.

## Methods

To compare the performance of working with maps which use different cartographic visualizations, we conducted an experiment with two tests (one for each of the two selected methods of visualization); for more details of the research design, see Fig 5. We applied a combination of confirmatory and exploratory data analysis methods [45, 47, 48].

The confirmatory analysis tested our hypotheses on the differences between extrinsic and intrinsic visualizations in map reading performance. The data collected were response time and correctness (as investigated by Elmer [43]). Several evaluation methods and concepts allow the measurement of user performance with an information system [49]. The most common parameters are effectiveness and efficiency. According to ISO 9241–11 [50], effectiveness is defined as the “accuracy and completeness with which users achieve specified goals”, and efficiency corresponds to the necessary resources (e.g., time) to achieve a desired result. We calculated effectiveness as the rate of correctness and efficiency as the task completion time [51]. The aim of the exploratory analysis of the eye-tracking data [52] was to gain deeper insight into the differences between the visualizations at the level of individual elements and to employ eye-tracking as a means of collecting objective data [53–55]. Eye-tracking is a valuable tool for studying eye behavior which occurs during map reading since it provides objective measurement of the visual strategies employed by map readers. The review article from Krassanakis and Cybulski [56] provides an overview of existing eye-tracking studies which have appeared in cartographic research over the last decade. The review showed that cartographers used eye tracking mainly in the evaluation of cartographic symbolization and design principles.

### Map and items design

**Fig 3:**
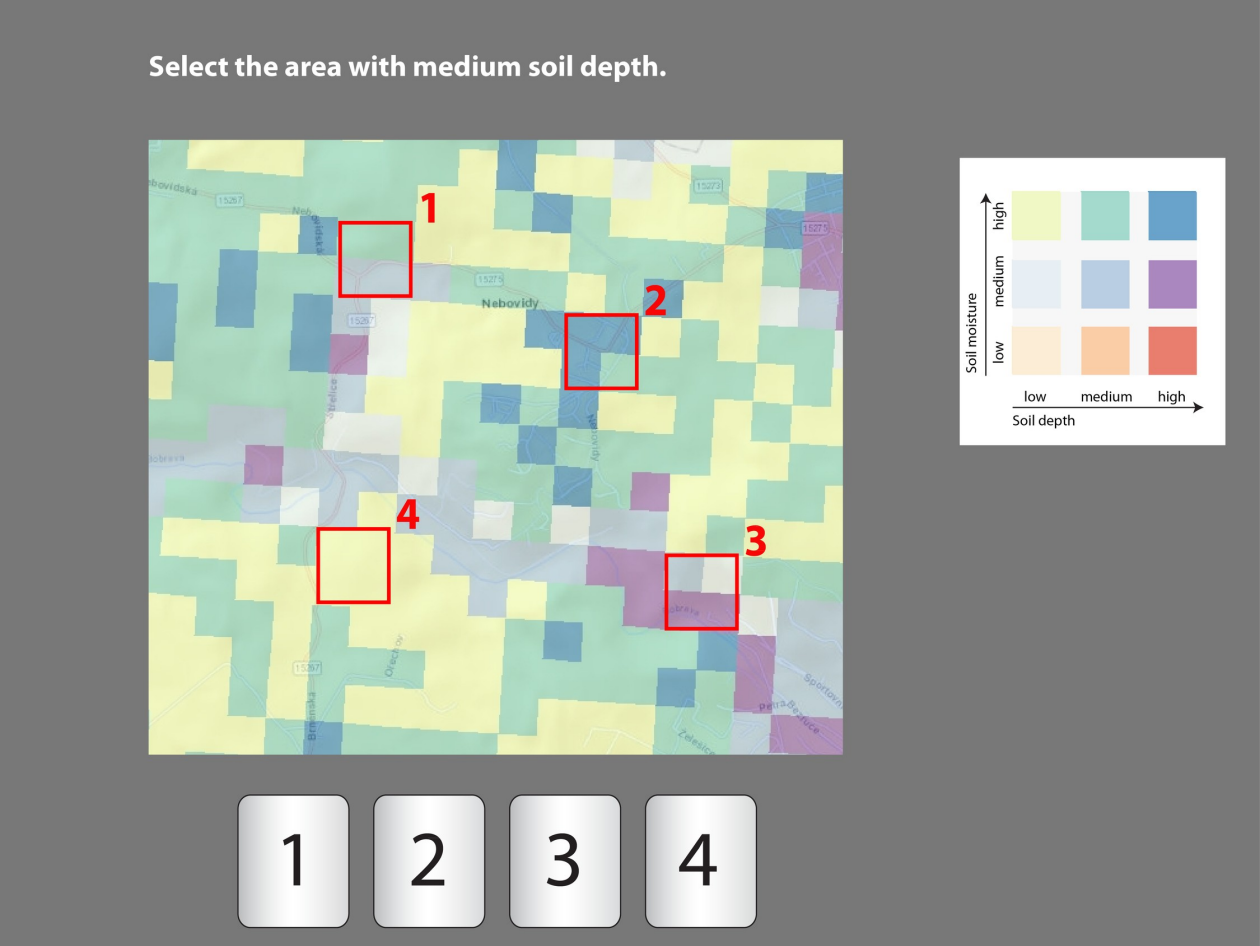
Example of an intrinsic visualization item (subtest A–part A.1.). The task was to select the area which contained “medium soil depth”; the correct answer was area No. 1.

**Fig 4:**
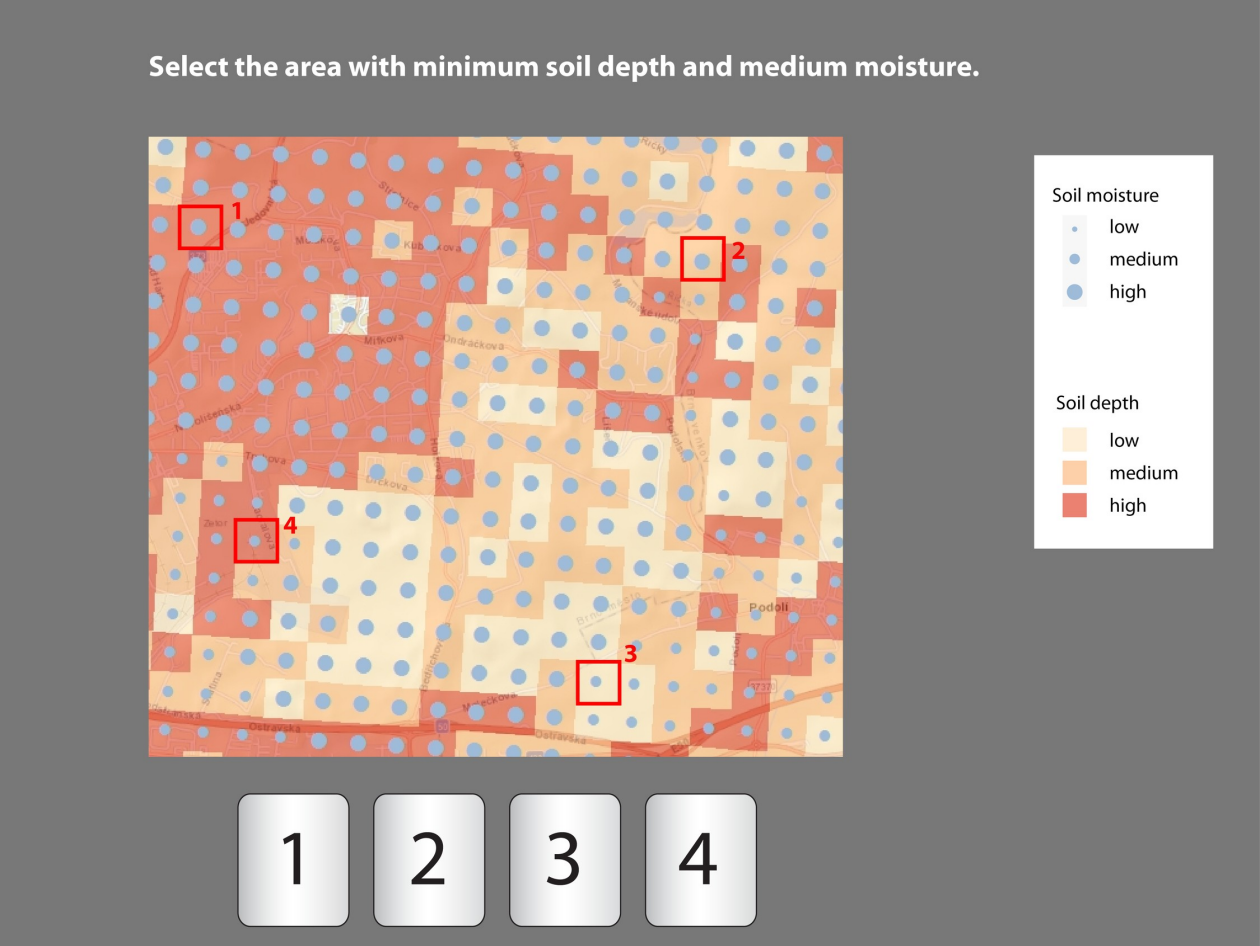
Example of an extrinsic visualization item (subtest B—part B.2). The task was to select the area which best satisfied the conditions of “low soil depth” and “medium soil moisture”; the correct answer was area No. 3.

The task layout was identical in both tests (Figs 3 and 4): instructions for the tasks were displayed in the upper area of the screen, the map legend was at the right, and the visual field of the map was in the center. The lower area of the screen displayed a button bar with four possible selections for the correct answer. The participants selected an area which satisfied the given condition (e.g., “Find the area with low soil moisture.”). In subtest A (Fig 3), the marked areas covered four square units; in subtest B, the marked area only covered one square unit (Fig 4). To answer the questions, participants were required to click on the correct button. Only one correct answer was possible.

We generated the visualization using ArcMap (version 10.7) using the color schemas from ColorBrewer 2.0 [57]. The extrinsic visualization used three circle sizes (6, 10 and 14 pts; #deebf7) to indicate soil moisture, and three color classes (#fee8c8, #fdbb84 and #e34a33) to indicate soil depth. The colors were selected to suit a realistic representation of the phenomena as they occurred in reality, such as blue for moisture and brown for soil depth. Three colors were used to create the intrinsic visualization (A: #e0f3db, #a8ddb5, #43a2ca; B: #e0ecf4, #9ebcda, #8856a7; C: #fee8c8, #fdbb84, #e34a33). A brown color scheme was used to indicate dry areas, and green-blue was used to indicate wet areas. Soil depth was indicated using a geographical principle, darker shades representing greater depth. All colors had a transparency of 40% to allow the base map to be visible. To create the base map, OpenStreetMap data was used [58].

### Procedure

The study was designed to illustrate the effect of various types of task. As mentioned in the introduction, we evaluated the maps / visualizations according to their purpose. We therefore designed the study to depict two types of phenomena. In the first scenario, the aim was to answer a question which related to only one variable (either soil moisture or soil depth). In the second scenario, participants were required to think about both phenomena in parallel. We assumed that the extrinsic method would be more suitable for an isolated assessment of phenomena because of its properties (both variables are presented separately through different visual qualities). The intrinsic method, however, is relatively more suitable for tasks which involve a unified search. Another reason for diversifying the task types (division into subtests 1 and 2) was to produce greater informative value and reliability in the achieved results. If performance of the extrinsic method possessed greater stability for each of the types in all tasks, the assumption that this method produces better results from the examined lay population would be more strongly supported.

**Fig 5:**
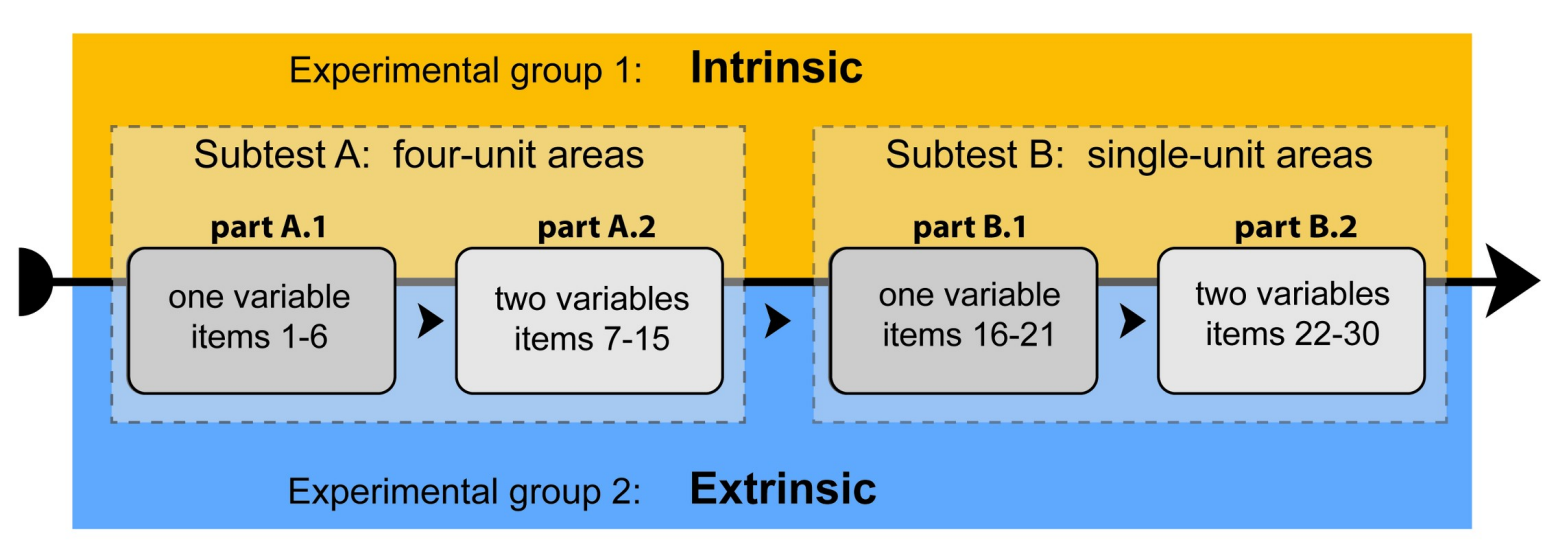
Between-subject experimental design (subtests A and B; parts A.1, A.2, B.1 and B.2). Both independent experimental groups, Intrinsic and Extrinsic, performed the test in exactly the same manner. The order of all items was constant for both groups, and all participants.

The test involved a total of 30 items. The first parts of each subtest (A.1 and B.1) contained six items which focused on a single phenomenon (Fig 3). The items covered six possible options: low, medium and high soil moisture, and low, medium and high soil depth. In the second parts of each subtest (A.2 and B.2), participants were asked questions about the two phenomena in each item item (Fig 4), with both A.2 and B.2 covering nine options (low moisture and low soil depth, low moisture and medium soil depth, etc.). We employed a between-subject design (Fig 5) to eliminate the effect of interference caused by experience with the given type of task.

Each participant completed an informed consent form, received a financial reward and was randomly allocated to one of the two research groups. Before the experiment, they were informed about the expected duration of the tests and given the opportunity to ask the experimenter questions. The instructions required the participants to work without interruption during the assessed part of the experiment. No participant required any additional explanation, and during a brief follow-up inquiry, no participant reported any problem in comprehending the content of the tasks. The participants received feedback on the correctness of their responses for the two sample items (one sample item was presented at the beginning of each subtest, A and B). No feedback was given during the assessed component of the tests. Each test item was preceded with a fixation cross displayed for 500 ms in the same position each time in the upper area of the screen.

### Apparatus

The test was administered using a DELL Precision M4800 notebook with a 22′′, 60 Hz AOC E2260P external monitor. The resolution was set at 4:3 (1024 x 768) to correspond exactly to the stimuli (Figs 3 and 4). The participants used a mouse to select their answers. The experimenter was present throughout the experiment to monitor its course. Mounted to the monitor was a remote SMI RED-m eye-tracker with a sampling rate of 60 Hz to collect eye-tracking data. Eye-tracking data collection, calibration and validation was done using the SMI Experiment Center 3.7 software. The calibration procedure was only considered satisfactory when the values returned by the eye-tracker were within 0.5°. The experiment was administered using the Hypothesis software tool [45, 53] (a web-based tool used in research and psychological diagnostics [59]). The behavioral raw data were exported from Hypothesis in “.xlsx” format and then processed using *R* (version 4.0.0) with the “rstatix” [60], “rcompanion” [61] and “multicon” [62] packages. Because of the relatively small sample size, we incorporated several specific procedures in our analyses. First, we used non-parametric statistical tests (i.e., Wilcoxon’s rank-sum test for independent samples and Wilcoxon’s signed-rank test for paired samples), which do not require Gaussian data distribution and can process potential outliers. Second, we reported not only the related effect sizes (i.e., rank-biserial correlation; *r*) but also their 95% confidence intervals (*CIs*), which were computed on the basis of 10,000 bootstraps. Third, we computed 95% *CIs* for the descriptive statistics of means and medians. This step gave us deeper insight into the obtained results, especially with respect to the small sample size since *CIs* tend to be very wide in small samples, and therefore for reliability, any potential significant differences should not be permitted to overlap. Eye-tracking data were imported into the OGAMA 5.0 software and paired with the behavioral data via HypOgama [53]. The fixations were calculated using the I-DT model with the parameters set to the following values (as recommended by Popelka et al. [53]): maximum distance = 20 px, minimum number of samples = 5; “do not merge consecutive fixations”.

### Participants

The Research Ethics Committee of Masaryk University approved this project (No.: 0257/2018). Participants were recruited via social networks and each signed an informed consent form. They received a financial reward (approx. 8 euros) for participation in the study.

The research sample was composed of 31 students (8 males and 23 females), aged between 19 and 28 (m = 21.8, med = 21). The sample was randomly divided into an “intrinsic” group and an “extrinsic” group (block randomization was used). The former (intrinsic) group consisted of 15 students (2 males and 13 females; m = 21.4). The extrinsic group consisted of 16 students, 6 males and 10 females (m = 22.3). All the participants were students of social sciences and humanities (Faculty of Arts or Faculty of Social Studies) at Masaryk University. Students of geography and related fields were excluded from the study.

The eye-tracking part of the study yielded 23 datasets; for the remainder of the participants (8), no data were recorded during the session for technical reasons. The data were from 4 males and 19 females, aged between 19 and 28 (m = 22.22, med = 22). The “extrinsic” group was composed of 12 students; the “intrinsic” group consisted of 11 students.

After completing the experiment, we performed a quality check of the eye-tracking data. The total data loss was 2.65% for the extrinsic group and 4.1% for the intrinsic group. All items with a dropout rate of above 10% were excluded from the analysis: this was 22 data points (out of a total 330 data points) in the case of the intrinsic method and 6 data points (out of a total 360 data points) in the case of the extrinsic method. No participant was excluded completely (because of a high dropout rate throughout the test).

## Results

We used several metrics which employ extrinsic and intrinsic methods of visualization to evaluate the differences between the groups in participant performance. We examined both behavioral (correctness, response time) and eye-tracking (dwell time, direct saccades) metrics. Details of the metrics calculations are specified in the respective section of the Results chapter. Non-parametric statistics were used to calculate the differences between and within the groups. A Wilcoxon rank-sum test was applied to compare independent groups (i.e., extrinsic vs. intrinsic); a Wilcoxon signed-rank test for dependent samples was used to compare performance between subtests. Effect size (r) was calculated for all results to determine the size of the differences [63].

A post-hoc sensitivity analysis of the differences between two independent means according to G*Power [64] (*1-β* = 0.80, *α* = 0.05, *n1* = 16, *n2* = 15, two-tailed) showed that with the given sample, we would only be able to detect medium to large effect sizes with differences between the two groups greater than a standard deviation of 1 (non-centrality parameter *δ* = 2.899, critical *t* = 2.8987, *df* = 29, *d* = 1.042). We therefore did not interpret any results with small effect sizes.

Split-half reliability coefficients performed on two random halves and adjusted with the Spearman-Brown prophecy formula were also calculated for each subtest. The results indicated that all the task subtests were reliable (mean of the split-half correlations for A1 = 0.847, A2 = 0.835, B1 = 0.889, and B2 = 0.736).

### Correctness

**Fig 6:**
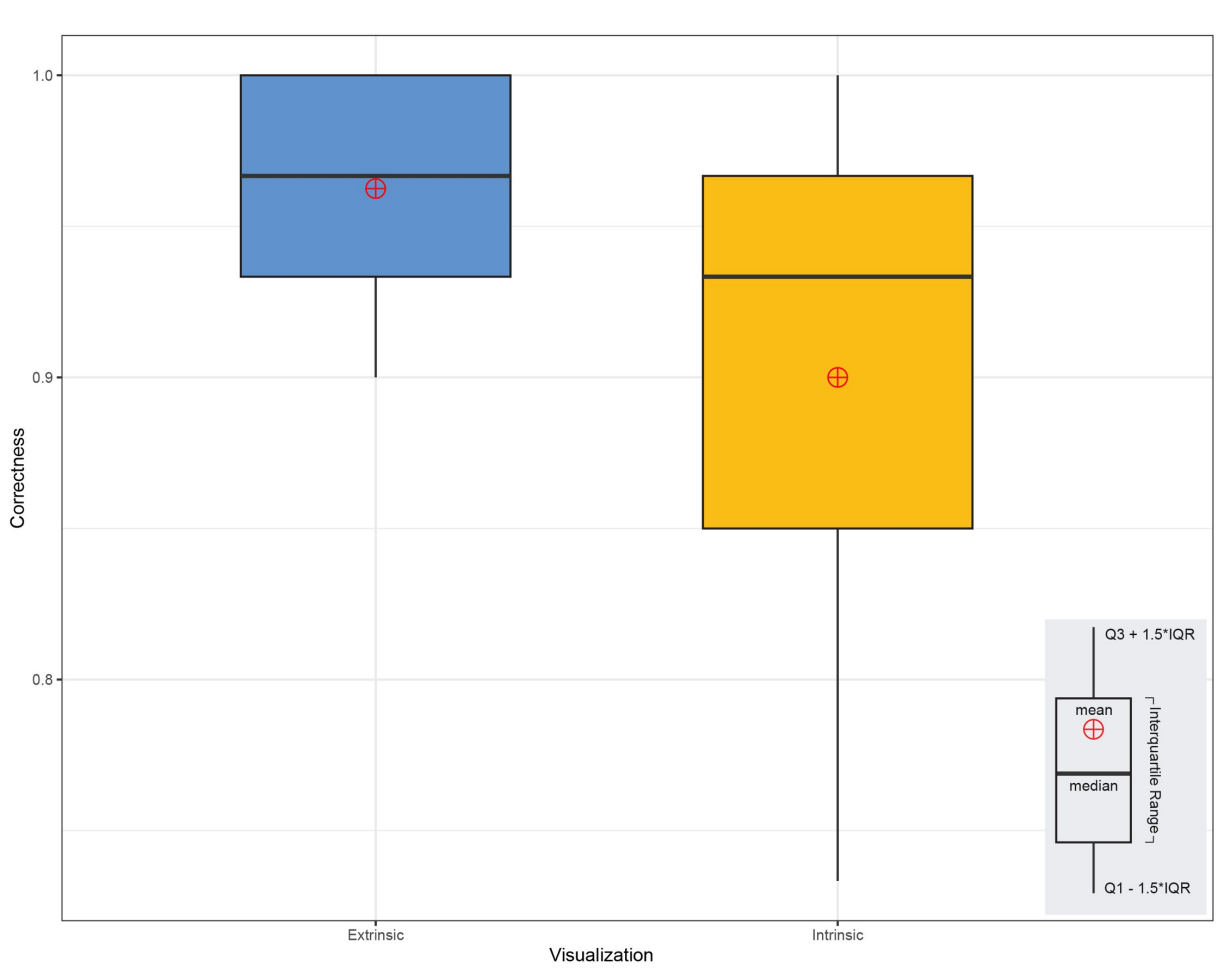
Response correctness for the entire test. Correctness was calculated as a ratio of the number of correct answers to the number of all answers.

Response correctness was one of the key parameters observed in the map-related tasks. Using the Wilcoxon rank-sum test, we compared the overall correctness of the responses related to the extrinsic and intrinsic groups. The extrinsic visualization showed a significantly higher overall correctness (N = 16, 96.3%) than the intrinsic visualization (N = 15, 90.0%), with a moderate effect size (Z = 173, *p* = 0.031, *r* = 0.390 [95% *CI*: .059, .656]). The results are charted in Fig 6.

**Fig 7:**
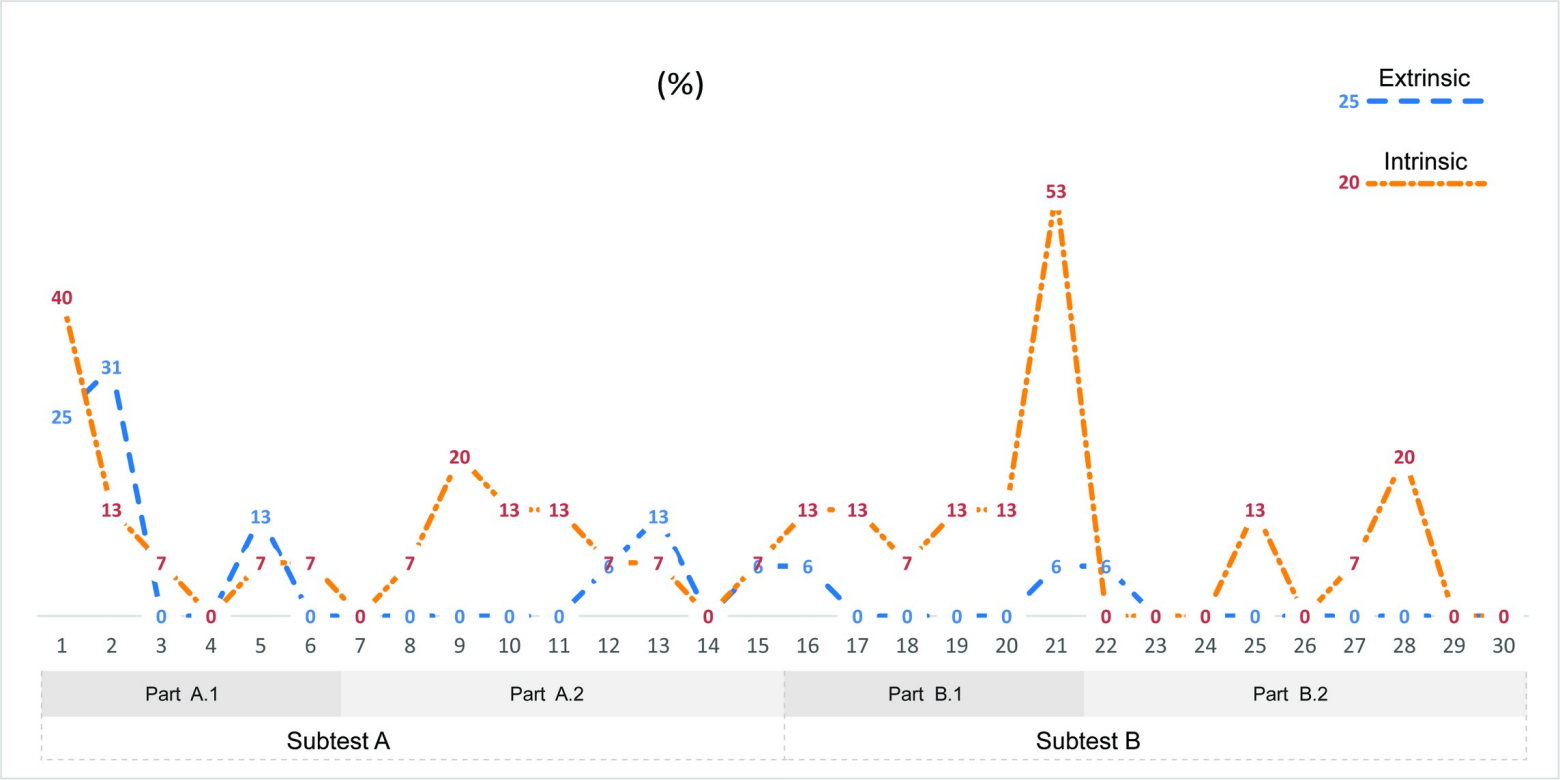
Error rate per item. INT–red/yellow, EXT–blue. Particular attention was given to items with a significant difference between the two visualizations (1, 2, 9, 21, 28). The error rate was calculated as a percentage of incorrect answers of all answers.

We also investigated incorrect responses to explore the error rate at the level of individual items (i.e., the distractors selected). Particular attention was given to items with a significant difference between the two visualizations, namely items No. 1, 2, 9, 21 and 28 (Fig 7). In the case of all items with the exception of No. 2, the intrinsic method was associated with higher error rates (item No. 2 showed a reverse scenario). A plausible explanation of the above phenomenon was identified only with respect to item No. 21 (Fig 7). The item required the participants to select the area with the lowest soil depth. The correct answer was unit No. 1 (lowest soil depth/highest moisture). In the intrinsic visualization, participants tended to select unit No. 3 (medium soil depth/medium soil moisture), which can likely be explained by unit No. 3 being surrounded by a darker color and thus appearing lighter (see [65–67]) and could therefore have been misinterpreted as the neighboring value (lowest soil depth/medium soil moisture). We identified no other trends.

### Response time

**Fig 8:**
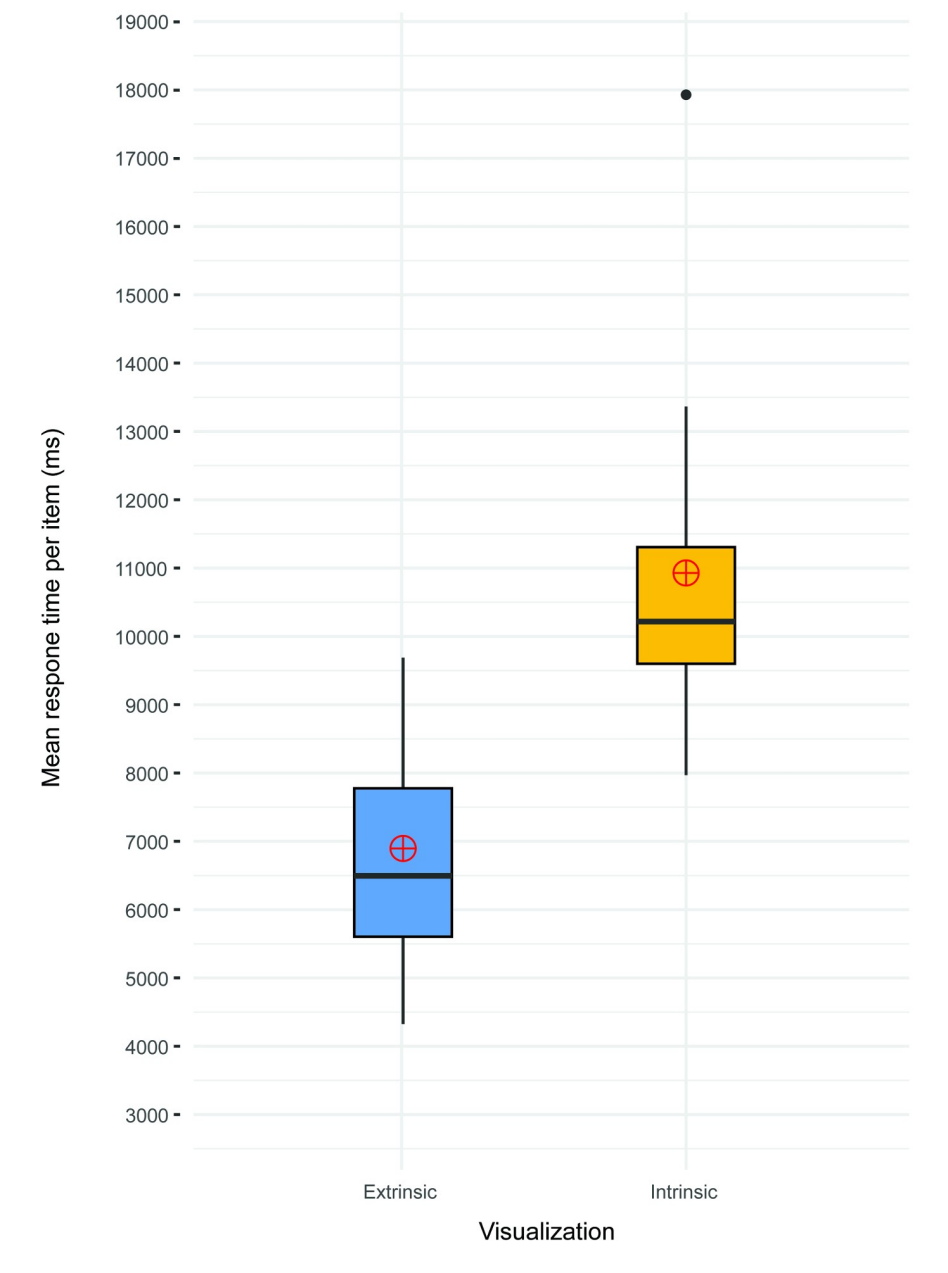
Mean response time per extrinsic/intrinsic visualizations (calculated from the response times to all extrinsic/intrinsic items for all participants).

For a comparison of processing speeds (response times; RTs), we applied the Wilcoxon rank-sum test. For each subtest, we performed a separate univariate outlier analysis. The analysis revealed three cases of extremely long and irregular response times (over 20,000 ms, different participants) and were excluded from further analysis. However, reaction times are usually distributed ex-Gaussian and demonstrate a rapid rise on the left and have a long positive tail on the right [68, 69]; the traditional outlier detections (e.g., ± 2 SD or 1.5 IQR) are therefore not recommended [70] since these extreme values should not be understood as outliers. Hence, we decided to keep the remainder of the outliers and applied non-parametric statistical analyses instead. The response time analysis therefore covered both correct and incorrect answers. The total response time was significantly less for the extrinsic method (N = 16, median = 6.494 ms [95% *CI*: 5536, 8726]) than for the intrinsic method (N = 15, median = 10.217 ms [95% *CI*: 9588, 11597]), with a large effect size (Z = 12, *p* < 0.001, *r* = -0.767 [95% *CI*: -0.844, -0.607]; see Fig 8 and Table 1).

**Table 1:** Response times for the individual subtest parts (ms).

	Extrinsic				Intrinsic				Wilcoxon rank-sum test		
part	mean [95% *CI*]	sd	median [95% *CI*]	iqr	mean [95% *CI*]	sd	median [95% *CI*]	iqr	Z	*p*-value	Effect size *r* [95% *CI*]
A1	6622 [5571, 7673]	1972	6406 [5094, 7286]	2007	13634 [12009, 15260]	2934	14917 [10800, 16056]	3436	5	*p* < 0.001	-0.779 [-0.839, -0.650] large
A2	8609 [7272, 9946]	2509	7827 [6749, 10230]	3066	10102 [8944, 11259]	2090	10184 [7912, 11915]	3237	71	0.093	-0.305 [-0.607, 0.043] moderate
B1	4945 [4266, 5623]	1273	4762 [3992, 6041]	1781	10798 [8928, 12668]	3377	9825 [8275, 14162]	4416	3	*p* < 0.001	0.831 [-0.853, -0.735] large
B2	6666 [5861, 7470]	1509	6638 [5132, 7708]	2159	8192 [6996, 9388]	2159	7738 [6542, 9604]	2359	68	0.041	0.369 [-0.643, -0.036] moderate
whole test	6896 [6035, 7756]	1615	6494 [2172, 5536]	2172	10929 [9576, 12281]	2442	10217 [9588, 11597]	1705	12	*p* < 0.001	-0.767 [-0.844, -0.607] large

The same pattern was observed in a comparison of the RTs of individual subtests. The extrinsic stimuli consistently indicated lower RTs than the intrinsic stimuli. We identified the largest differences between visualizations in parts A1 and B1; the differences between visualizations in parts A2 and B2 were moderate. All the differences, with the exception of those related to A2, were significant at a significance level of 5% (Table 1). All the differences, with the exception of those related to A2 and B2, yielded large effect sizes; we also observed large gaps in the upper bounds in the confidence intervals of the extrinsic group and the lower bounds of the confidence intervals in the intrinsic group, suggesting that the obtained statistically significant results were reliable.

**Fig 9:**
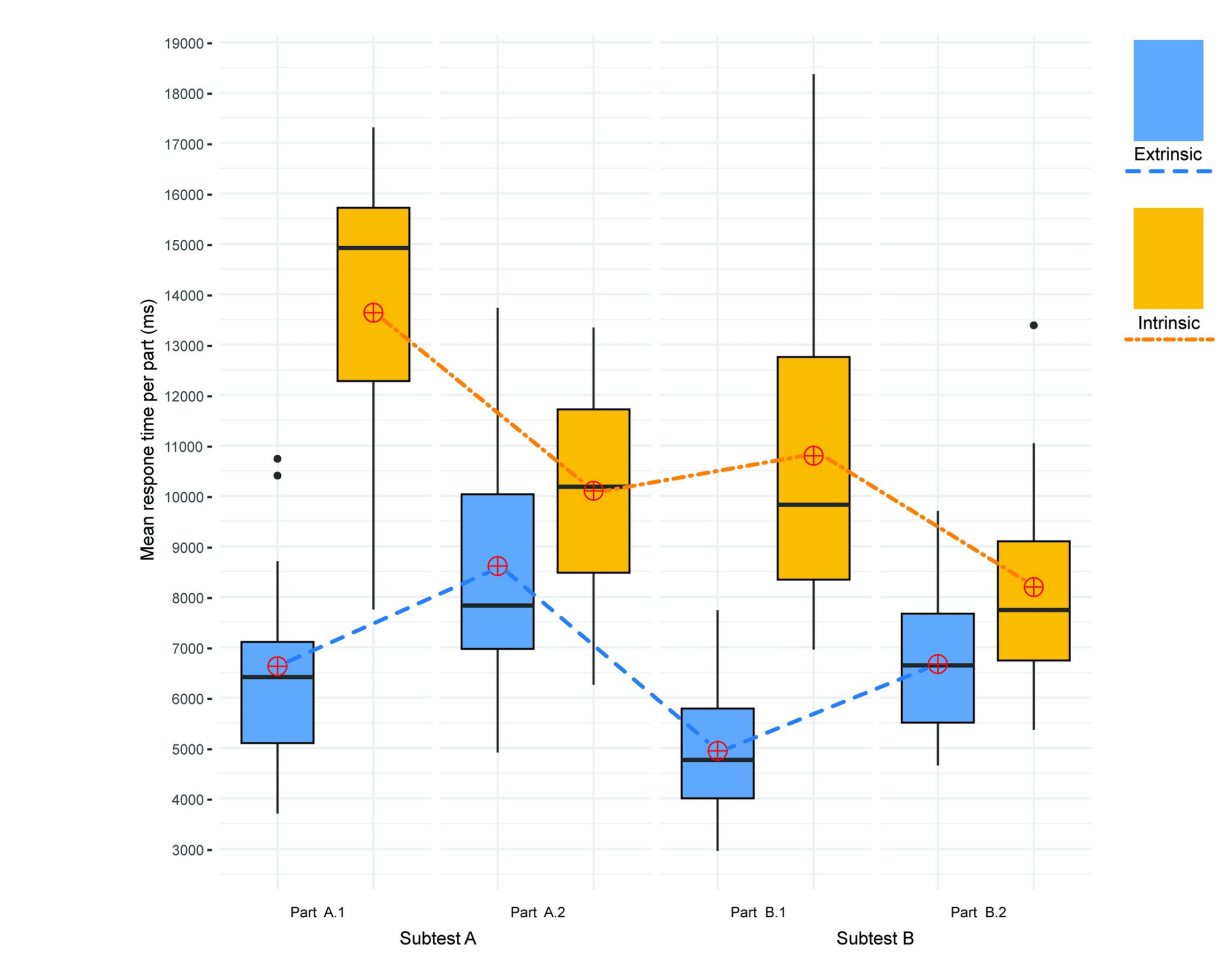
Mean response time (ms) per item (calculated for the individual subtest levels).

At the individual subtest levels (A1, A2, B1, B2), we examined the differences between the test items with one and two variables using the Wilcoxon signed-rank test. An exploration of response times at the subtest level revealed an interesting pattern (Fig 9). In the extrinsic “A” levels, A2 (two variables) resulted in significantly longer response times than A1 (one variable), with a large effect size (Z = 7, *p* < 0.001, *r* = -0.789 [95% *CI*: -0.880, -0.558]). We observed a similar effect in relation to the extrinsic “B” levels, where B2 showed significantly longer response times than B1 (Z = 0, *p* < 0.001, *r* = -0.880 [95% *CI*: -0.882, -0.879]). However, we noted an inverse pattern in relation to the intrinsic visualizations, where A1 (one variable) received significantly longer response times than A2 (large effect size; Z = 68, *p* = 0.021, *r* = 0.655 [95% *CI*: 0.227, 0.886]), and similarly, B1 resulted in significantly longer response times than B2 (Z = 115, *p* < 0.001, *r* = 0.806 [95% *CI*: 0.589, 0.883]). In a comparison of the effect sizes for both the extrinsic and intrinsic visualizations, we can see that the effect sizes of the differences between one and two variables were greater in the extrinsic group. It can therefore be assumed that extrinsic visualization is more efficient when a single variable is applied, while intrinsic visualization is more suitable for two variables.

**Fig 10:**
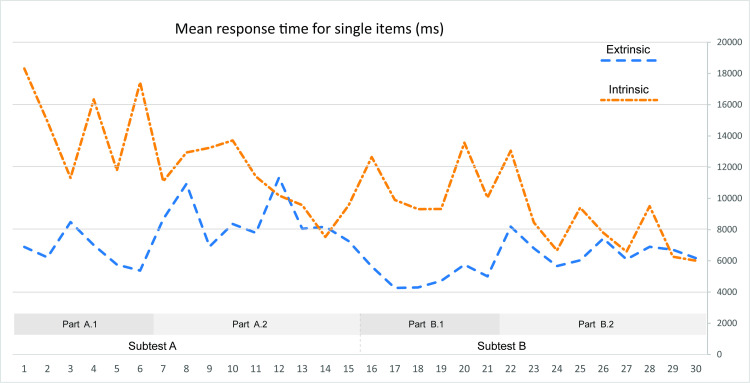
Mean response times (ms) per individual items for extrinsic and intrinsic visualization (all items).

In addition to the above, we performed a response time comparison at the item level. For most items, extrinsic visualization resulted in shorter response times than intrinsic visualization. The opposite was true for only four items, intrinsic visualization only inducing slightly shorter response times (Fig 10). The differences were significant for most items. An analysis at the item-level also revealed two other interesting phenomena: the first consisted in significant variability across the items observed, even within the individual subtests. This variability reflected the complex nature of maps as research stimuli. The difficulty of a test item depended on an array of interacting factors, including the type of correct answer, the distractors selected and the visualized territory. The second observed phenomenon was that the obtained performance curves associated with both visualization types did not overlap, meaning that the difficulty of the test items varied depending on the type of visualization. In other words, the items that were relatively simple to solve in combination with intrinsic visualization were more difficult with extrinsic visualization, and vice versa.

### Eye-tracking analysis

For the purposes of the eye-tracking analysis, the stimuli were divided into three key Areas of Interest (AOI): instructions (the textual component), map legend and map. The analysis consisted in a comparison of the dwell times related to the AOI of the individual items (Fig 10 and Table 2). We were also curious about a comparison of the total dwell times for the extrinsic (N = 12) and intrinsic (N = 11) groups (see S1 File). The results showed significant differences in total dwell times, the extrinsic visualization indicating shorter dwell times with a large effect size. A closer examination revealed that the differences were caused by map legend dwell times. The “extrinsic” group displayed significantly shorter dwell times on the map legend than the intrinsic group, with a large effect size and also with a very large gap between the upper bounds of the confidence intervals of the extrinsic group and the lower bounds of the confidence intervals of the intrinsic group. No significant differences were observed in the dwell times during the instructions.

**Table 2:** Summary of AOI dwell times (ms).

	Extrinsic				Intrinsic				Wilcoxon rank-sum test		
AOI	mean [95% *CI*]	sd	median [95% *CI*]	iqr	mean [95% *CI*]	sd	median [95% *CI*]	iqr	Z	*p*-value	Effect size *r* [95% *CI*]
Instructions	2514 [2172, 2856]	539	2570 [2193, 2703]	470	2566 [1807, 3325]	1130	2350 [1579, 4252]	1366	74	0.6505	-0.103 [-0.338, 0.53]
Map Legend	216 [84, 348]	208	180 [62, 246]	127	3740 [2921, 4559]	1219	3675 [2411, 4864]	1841	***0***	***p < 0*.*001***	***-0*.*847 [-0*.*851*, *-0*.*749]***
Map	3773 [3048, 4497	1141	3592 [2683, 5117]	1785	3769 [2895, 4642]	1300	3188 [2771, 5847]	1354	70	0.833	- 0.051 [-0.363, 0.49]
All AOI	6503 [5549, 7457]	1502	5970 [5052, 8326]	2712	10075 [8350, 11800]	2567	9142 [8014, 13853]	2192	***10***	***p < 0*.*001***	***-0*.*719 [-0*.*842*, *-0*.*475]***

**Fig 11:**
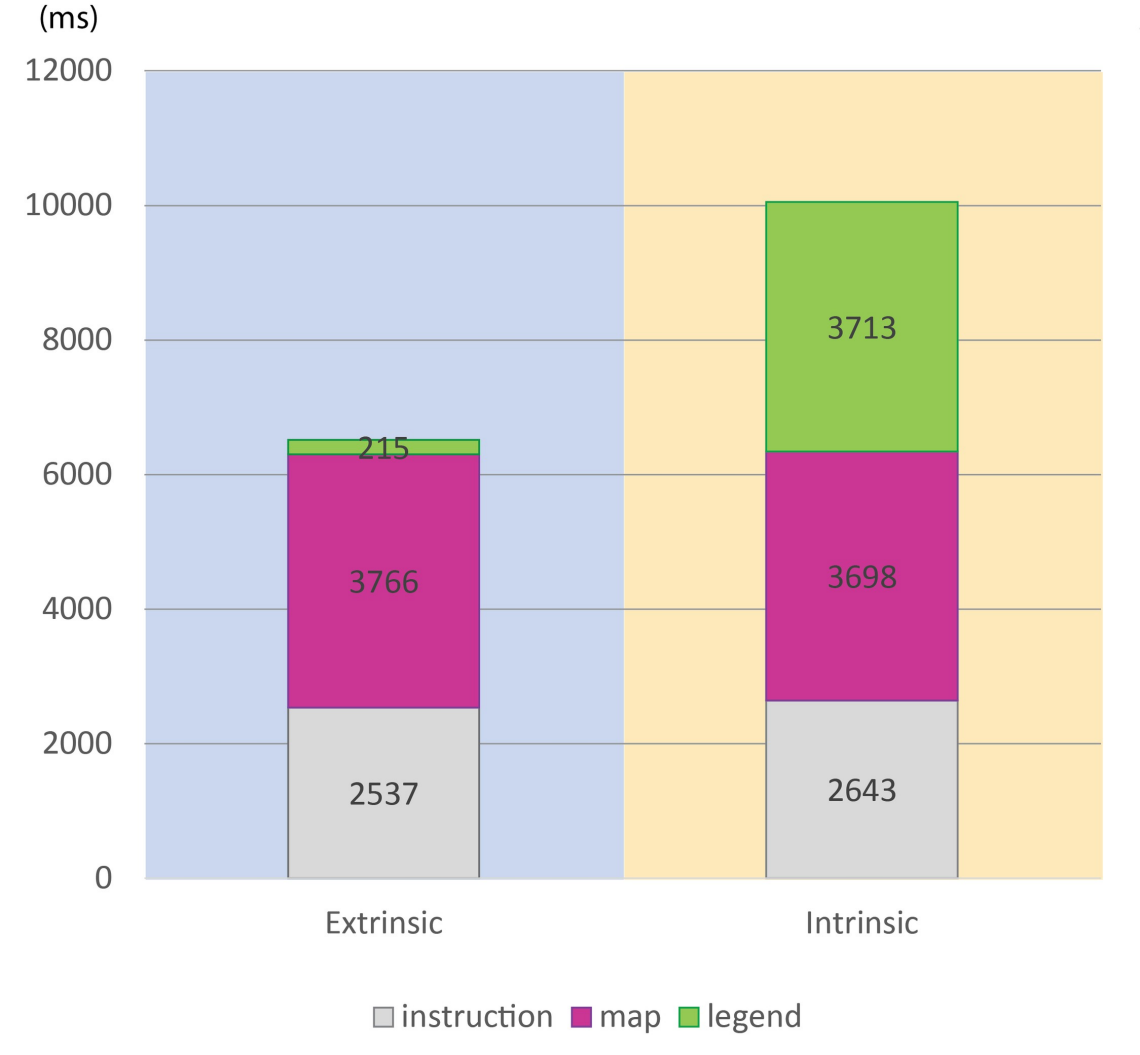
Mean AOI dwell time per extrinsic/intrinsic group (ms).

**Fig 12:**
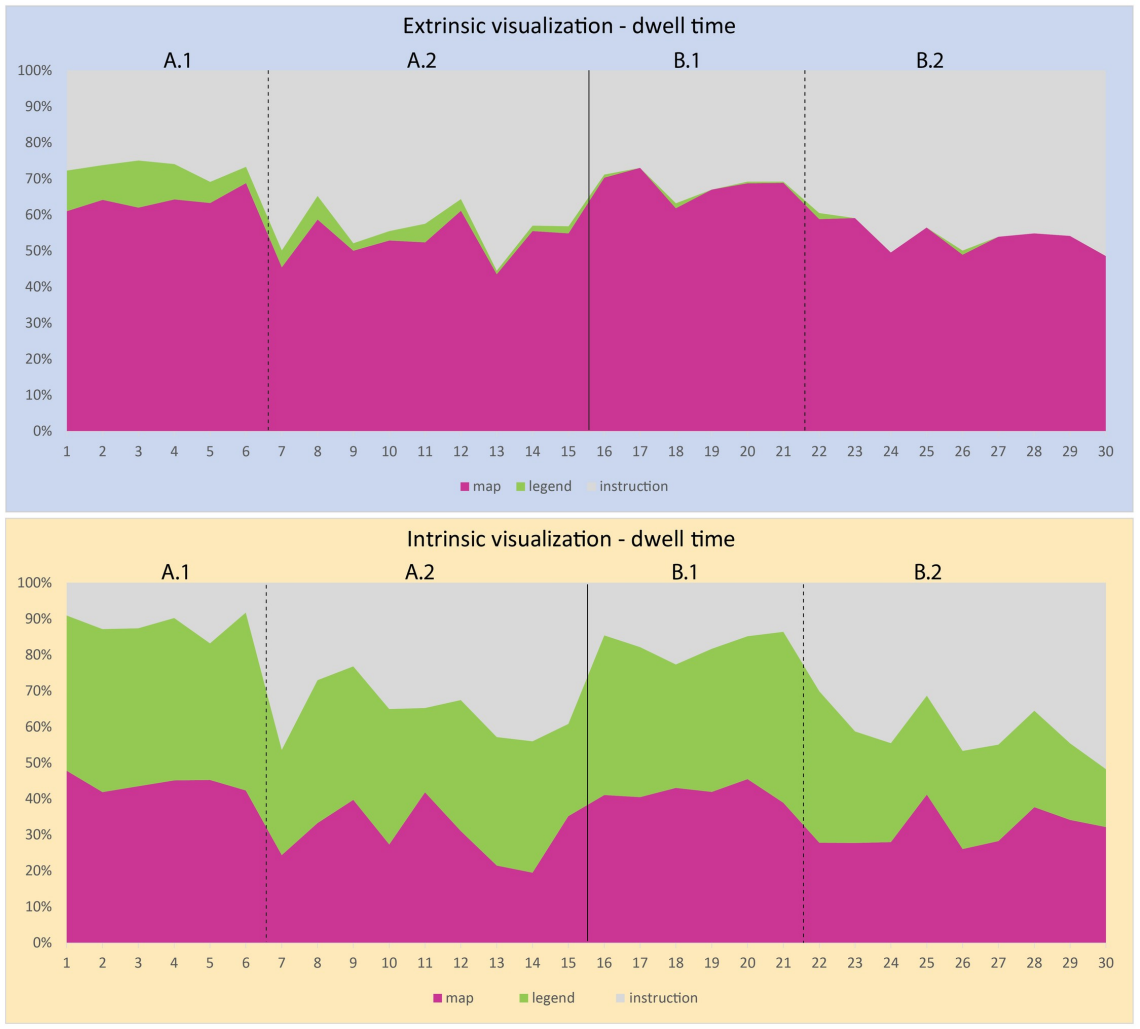
**Dwell time on AOI (%) for the extrinsic visualization (top) and intrinsic visualization (bottom).** Extrinsic visualization–proportion of dwell time at AOI in single items; (top); Intrinsic visualization–proportion of dwell time at AOI in single items (bottom).

We also visually inspected the oculomotor data in this study at both the item and subtest levels. The times spent on AOI were converted into percentages. The graphs in Figs 11 and 12 show the ratio of time spent on the instructions, map and map legend. We can observe that at the beginning of the experiment, the participants in the extrinsic group needed approximately 10% of the total time-on-task to decode the map’s legend; as their experience increased, the time needed to decode the map legend decreased to as little as zero for some items. The “intrinsic” group, by contrast, initially spent about 40% of the time exploring the map legend, with the percentage decreasing with experience, although it remained relatively high (30%).

**Fig 13:**
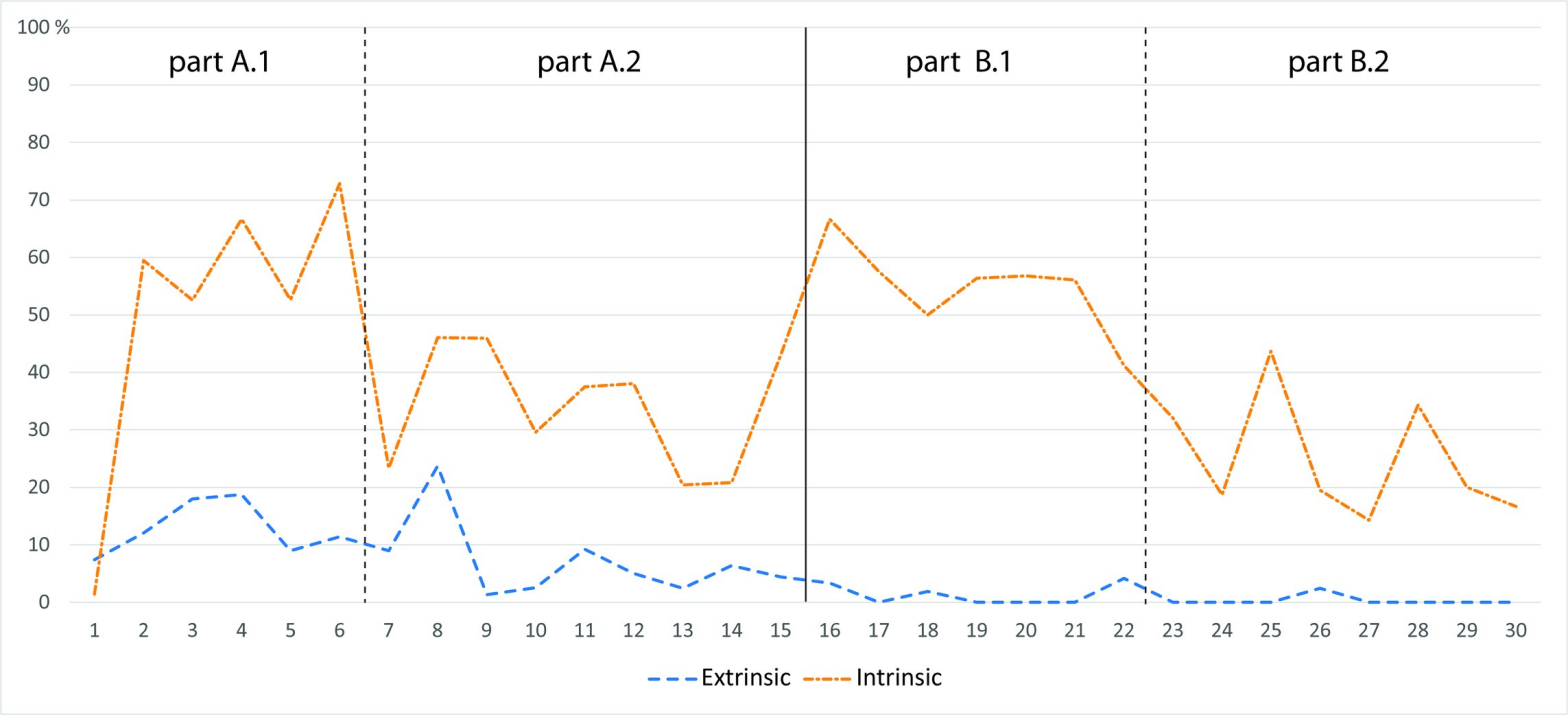
Ratio of the direct map-to-legend/legend-to-map saccades to the total number of direct saccades (%) between the defined AOI (instructions, legend, map, button bar).

A comparison of direct saccades (transitions) between the map legend and the visual field of the map reveal a pattern similar to that described for dwell times. Four AOI were defined (instructions, map, map legend, button bar), and a matrix of transitions between the AOI for each item was generated. Fig 13 displays the ratio of the direct map-to-legend/legend-to-map transitions to the total number of transitions between the AOI. It is clear from the graph that the “extrinsic” group only made use of the map legend at the beginning of the experiment; later, direct saccades occurred less. The “intrinsic” group, by contrast, made use of the legend throughout the tasks, with the number of repeated map-to-legend transitions being higher for the tasks with a single variable (A1 and B1).

## Discussion

The results of the present study showed that the intrinsic visualization employed was significantly less effective and efficient than extrinsic visualization. In the case of intrinsic visualization, the participants needed significantly more time to solve the tasks and simultaneously produced more errors.

Nevertheless, the response time differences between the two visualization methods were less pronounced when two variables were considered (soil moisture and soil depth). This levelling was caused by the increase in the time needed to solve the tasks with two variables in both extrinsic subtests (A and B). The effect was not observed with the intrinsic visualization. The above finding is in accordance with the studies performed by Nelson [39] and Elmer [43]. We emphasize that the findings and differences between the visualizations can be generalized only with regard to the population on which the research was conducted. It is a lay population with a basic level of map skills and who may also achieve higher education in humanities and social sciences. Conversely, as the results of the study [6] suggest, a population with a higher level of map literacy may prefer the intrinsic method in certain tasks. Another potentially significant change which affects how we work with maps is the type of formal education or the cultural background of users [71–73].

An exploratory analysis of eye-tracking data provided a deeper insight into the above results. Dwell time analysis showed that both groups spent comparable time on the instructions and the map; the reason for longer response times of the “intrinsic” group consisted in the time needed to decode the map legend. While the “extrinsic” group took only a fraction of the total dwell time to interpret the map legend, in the case of the “intrinsic” group, it was over a third of the total time-on-task. An analysis at the item level revealed yet another tendency: at the beginning of the experiment, the participants in the extrinsic group needed approximately 10% of the total time-on-task to interpret the map legend; as their experience increased, this time decreased to as little as zero for some items. The “intrinsic” group initially spent about 40% of the time decoding the map legend, and although this percentage decreased with experience, it remained as high as 30%. The above results appear to indicate that the map legend of an intrinsic visualization is so complex and essential that it needs to be referred to throughout the task. The same conclusion could be drawn from an analysis of direct saccades between the defined AOI.

A comparison of the performance of the “extrinsic” and “intrinsic” verified the greater effectiveness and efficiency of extrinsic visualization. The results also showed that the type of task (i.e., whether it concerned a single variable or two variables) had a definitive effect on performance, which is in accordance with the statement [e.g., 7, 6] that the performance of map work partly depends on whether the given type of visualization is suitable for the task at hand. If we want to understand the effects of different forms of visualizations during the process of cartographic communication, we must first understand the underlying cognitive processes [17–20]. A particular type of task may require the activation of specific cognitive processes which are appropriate to a particular visualization type. Anderson [74] emphasized that visual representations differ not only in the coding system, but, importantly, in the cognitive processes they evoke.

The results of the present study indicate that in the case of extrinsic visualization, the map user first perceives and processes both visually distinct variables consecutively, subsequently “putting them together” in their working memory when solving the task. Intrinsic visualization, by contrast, requires only one variable to be kept in working memory at any moment when the task concerns two variables (soil moisture and depth). When the task involves a single variable, however, the user must first decode the map legend and keep all three levels of the variable in their working memory. In the above, the results confirm our assumption that the cartographic visualization must be selected according to the type of task or operation to be performed with the particular map.

Our study is not without limitations. One of the limitations was the small sample size and resulting low power in the statistical tests. Low power may lead to an increase of the risk that the existing differences in performance will be falsely not detected as statistically significant. However, we took several (mostly statistical) precautions to prevent the misinterpretation of our data. We conducted a post-hoc sensitivity analysis which suggested that with a given sample size, medium to large effect sizes could be acceptably interpreted (see the first section of the Results chapter), whereas results with small effect sizes would be inconclusive. Rigorous statistical procedures which allow the interpretation of results on smaller sample sizes were also employed in the study (including bootstrapped confidence intervals for means, medians and effect sizes). Regarding the research sample’s composition, we attempted to form a sample which was as homogenous as possible (age, level of education, field of study, experience with maps, etc.) and randomly added participants to the extrinsic/intrinsic groups to obtain an equally balanced sample size for each experimental condition (block randomization) and to reduce potentially confounding effects.

Furthermore, the sample size in our study does not deviate from the standard practice of the field of research in question. King [52] pointed out that many studies work with the relatively small samples given by the high requirements for laboratory equipment. Cognitive cartography surely is one of the fields in which certain studies have contributed significantly to increasing knowledge, regardless of their sample sizes [75–78].

However, the size of the research sample and its composition (European university students of humanities and social sciences with common map literacy skills) permitted us to generalize the conclusions for similar populations. Further research with this method on different samples is required to expand the results of the present study and explore how different population characteristics (map literacy, level and type of formal education) affect the preference for specific types of visualization.

## Conclusion

The performed confirmatory analysis verified the superiority of extrinsic visualization in the case of a population of individuals with higher formal education in humanities and social sciences, both in terms of effectiveness and efficiency. Complementary exploratory analysis of eye tracking data suggests that the reason is the character of the intrinsic map legend, which demands greater cognitive resources from map readers during processing. The present study’s findings are significant not only for basic research in visualization and cognitive processes but also in their implications for cartographic practices. Even despite a relatively small sample size, the results were statistically significant, but also, importantly, very large effects were discovered. The extrinsic method can be considered convincingly proved as a more suitable visualization type for the given types of task and the lay population.

The results of the present study also raise the question of whether a higher level of efficiency and effectiveness would be maintained with the extrinsic method even if the target population was composed of individuals with high levels of map literacy and different formal education, and whether cultural background plays a role. The specific character of a cartographic visualization sets a significant limit on empirical testing. Even a relatively minor change in partial parameters (e.g., the absolute size of the circles in the case of the extrinsic method, or using a different color scheme in case of the intrinsic method) may affect, for example, the processing speed of visual search or the memorability of the legend, and consequently result in a difference in overall performance. Therefore, to maintain research rationality, it seems reasonable to conduct more partial studies with relatively smaller samples while comparing a wider variability in the applied visualizations and their partial modifications. That is also our objective for future research: if the trends we uncovered are confirmed in studies which examine modified legends, the findings may then be generalized to a wider population, and the principle of the varying methods which were applied can be verified as the cause of the differences in processing. Changes in visualization parameters may also explain the revealed differences at the level of individual items.

## Supporting information

S1 File (DOCX)

S1 Fig (TIF)
