# Supplementary material for: A comparison of the performance on extrinsic and intrinsic cartographic visualizations through correctness, response time and cognitive processing
Source: PLoS One. 2021 Apr 21;16(4):e0250164. doi: 10.1371/journal.pone.0250164 (PMC8059811; doi:10.1371/journal.pone.0250164)
Supplement: S1 File — (DOCX) [file pone.0250164.s001.docx]

S1 File

The datasets relevant to the paper “A Comparison of the Efficiency of Extrinsic and Intrinsic Cartographic Visualizations through Correctness, Response Time and Cognitive Processing” are available from the:

https://osf.io/2t4ms/files/
