# Supplementary figures and images for: A comparison of the performance on extrinsic and intrinsic cartographic visualizations through correctness, response time and cognitive processing
Source: PLoS One. 2021 Apr 21;16(4):e0250164. doi: 10.1371/journal.pone.0250164 (PMC8059811; doi:10.1371/journal.pone.0250164)

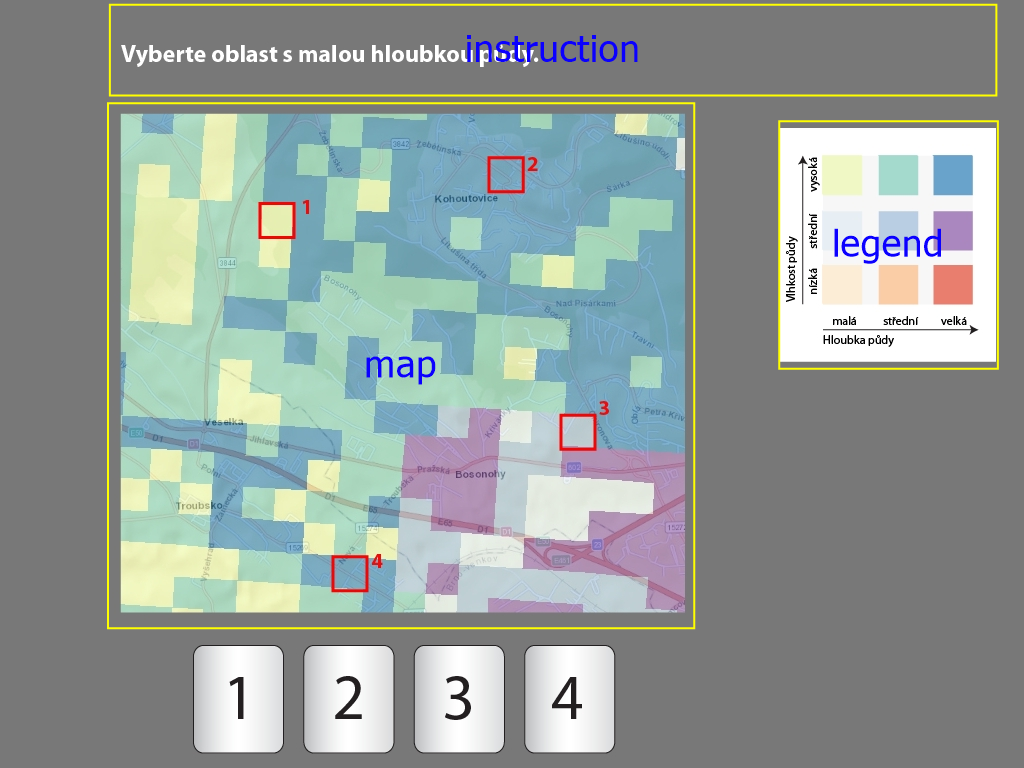

Supplement: S1 Fig — (TIF) [file pone.0250164.s002.tif]
